# Supplementary material for: A neutron tomography study: probing the spontaneous crystallization of randomly packed granular assemblies
Source: Sci Rep. 2018 Dec 5;8:17637. doi: 10.1038/s41598-018-36331-1 (PMC6281579; doi:10.1038/s41598-018-36331-1)
Supplement: Supplementary file 3 — A neutron tomography study: probing the spontaneous crystallization of randomly packed granular assemblies [file 41598_2018_36331_MOESM3_ESM.pdf]

Supplemental Material

A neutron tomography study: probing the spontaneous crystallization of randomly packed granular assemblies

Indu Dhiman[1,*], Simon A. J. Kimber[1], Anita Mehta[3], Tapan Chatterji[2,*]

[1]Neutron Scattering Division, Oak Ridge National Laboratory, Oak Ridge, Tennessee 37831, USA.
[2]Institut Laue-Langevin, 71 Avenue des Martyrs, 38000 Grenoble, France.
[3]Max Planck Institute for Mathematics in the Sciences, Inselstrasse 22, 04103 Leipzig, Germany.

*Correspondence: dhimani@ornl.gov, chatterji@ill.fr

Video_180Hz_06mm

Video_180Hz_06mm_1

Figure S1: Videos display the structural evolution of different phases obtained using adaptive common neighbor analysis at 180 Hz frequency and 0.6 mm shaking amplitude for two different orientations. Green and red colored spheres represent face centered cubic and hexagonal close packed clusters, respectively. While, the blue ones correspond to body centered cubic type and the white colored spheres are the ones with undefined coordination, where the local environment of the spheres may not resemble any of the known crystal structures close enough.
